# Modelling the impact of non-pharmaceutical interventions on the spread of COVID-19 in Saudi Arabia

**DOI:** 10.1038/s41598-022-26468-5

**Published:** 2023-01-16

**Authors:** Yehya Althobaity, Michael J. Tildesley

**Affiliations:** 1 The Zeeman Institute for Systems Biology and Infectious Disease Epidemiology Research, School of Life Sciences and Mathematics Institute, University of Warwick, Coventry, UK; 2 Department of Mathematics, Taif University, Taif, Kingdom of Saudi Arabia

**Keywords:** Computational biology and bioinformatics, Ecology, Zoology, Diseases, Health care, Mathematics and computing

## Abstract

Countries around the world have implemented a series of interventions to contain the pandemic of coronavirus disease (COVID-19), and significant lessons can be drawn from the study of the full transmission dynamics of the disease caused by—severe acute respiratory syndrome coronavirus 2 (SARS-CoV-2)—in the Eastern, Madinah, Makkah, and Riyadh regions of Saudi Arabia, where robust non-pharmaceutical interventions effectively suppressed the local outbreak of this disease. On the basis of 333732 laboratory-confirmed cases, we used mathematical modelling to reconstruct the complete spectrum dynamics of COVID-19 in Saudi Arabia between 2 March and 25 September 2020 over 5 periods characterised by events and interventions. Our model account for asymptomatic and presymptomatic infectiousness, time-varying ascertainable infection rate, and transmission rates. Our results indicate that non-pharmaceutical interventions were effective in containing the epidemic, with reproduction numbers decreasing on average to 0.29 (0.19–0.66) in the Eastern, Madinah, Makkah, and Riyadh region. The chance of resurgence after the lifting of all interventions after 30 consecutive days with no symptomatic cases is also examined and emphasizes the danger presented by largely hidden infections while switching control strategies. These findings have major significance for evaluating methods for maintaining monitoring and interventions to eventually reduce outbreaks of COVID-19 in Saudi Arabia in the future.

## Introduction

Emergence of the novel human coronavirus SARS-CoV-2 resulted in the fifth documented pandemic and is the third zoonotic human coronavirus that has emerged in the current century, after the severe acute respiratory syndrome coronavirus (SARS-CoV) in 2002 that spread to 37 countries and the Middle East respiratory syndrome coronavirus (MERS-CoV) in 2012 that spread to 27 countries^1^. A new severe outbreak of respiratory disease appeared for the first time in Wuhan, in December 2019^2^. The Huanan Seafood Market in Wuhan is believed to be the source of the epidemic in Wuhan, given that 55% of the first 425 confirmed cases were related to the marketplace^3^. Meanwhile, recent studies indicated a 96% similarity of the genetic sequences of this virus and bat coronaviruses^4^.

**Figure 1:**
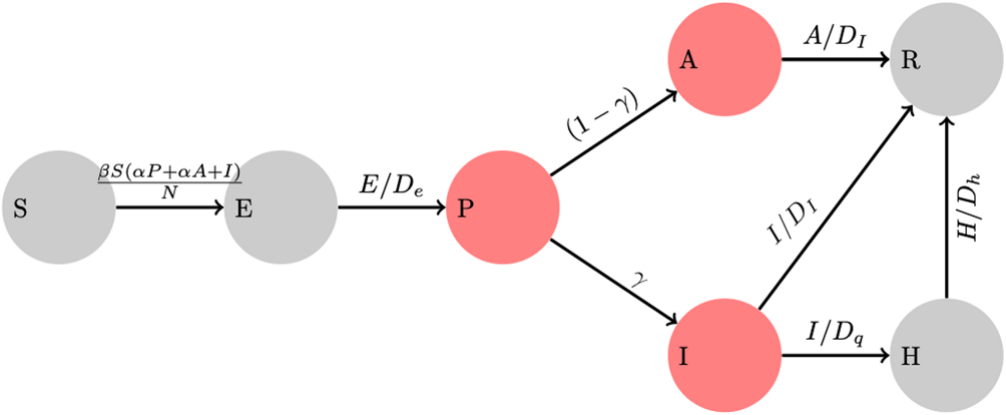
Schematic of the SEPAIHR model. Here we include seven compartments namely: susceptible (S), exposed (E), presymptomatic infectious (P), infectious (I), asymptomatic infectious (A), isolation in hospital (H), and removed (R). Two parameters of interest are $\gamma$ (documented infection rate, defined as the probability that a patient would be laboratory confirmed) and $\beta$ (transmission rate, defined as the number of individuals that a documented infected case can infect per day), which are considered to vary across time periods. In this model, the asymptomatic compartment A represents individuals who were not documented but could still transmit the virus to susceptible individuals.

In Saudi Arabia, the Ministry of Health confirmed the first case on 2nd March 2020—a Saudi citizen returning from Iran via Bahrain^5^. Following this introduction, the epidemic spread rapidly in Saudi Arabia and by 25th September 2020, the kingdom had reported 325509 cases, the highest among the Arabian Gulf countries^6^. After the confirmation of within-country human-to-human transmission, Saudi authorities were gradually implementing measures in order to understand the severity of the disease and to govern the geographical spread of the disease. These measures were accompanied by a series of non-pharmaceutical interventions (NPIs) at the level of the country and regions. These non-pharmaceutical interventions included the suspension of all travel into and out of Saudi Arabia, the compulsory wearing of masks in public places, the cancellation of social gatherings, and the home isolation of individuals with presumed infections. In addition, travel restrictions were implemented^5,7^. On 25th March 2020, the Saudi government enacted further control measures, including the isolation of all patients, those suspected of contracting the virus, and their close connections in an effort to prevent household and community spread. In addition, region-wide testing took place between 12th May and 25th September by designated health care workers and volunteers, to determine previously undetected symptomatic cases^8^. These interventions together with improved medical resources resulted in a flattening of the epidemic curve and reduced the effective reproduction number from 6 to 1 as well as the rate of spread throughout the country^7,8^.

Throughout the pandemic, mathematical modelling has been used as a tool to support government decision-making. Yang et al.^9^ developed a mathematical model to investigate the impact of quarantine and subsequent relaxation on the COVID-19 outbreak, and the model included two subpopulations based on different death rates in the young (59 years or less) and old (60 years or more). This model was developed to illustrate the epidemiological situation associated with the isolation and release of intermittent pulses in Brazil. In the UK, Keeling et al.^10^ proposed a deterministic model that divided the population into susceptible, exposed, documented infectious, undocumented infectious, and removed states, based on data on confirmed cases to give up-to-date predictions on the epidemic spread in ten regions of the UK. With the use of a global metapopulation epidemic model, Imai et al.^11^ investigated the size of the disease outbreak in Wuhan, with a focus on the spread of the virus from human to human. Their work concluded that strict control measures were necessary in order to reduce the effective reproduction number below 1. Tang et al. in^12^, proposed a mathematical model to predict the evolution of SARS-CoV-2 and evaluated the impact of governmental decisions on this evolution, with the goal of explaining the long duration of the pandemic in the 26 Brazilian states and their capitals, as well as in the Federative Unit of Brazilian states and capitals. Based on the rate of increase in new cases over a steady time, projections were carried out, and the graphics were displayed together with the most important governmental choices in order to analyze their influence on the epidemic curve in each Brazilian state and city^13^.

In this study, we employed an extended SEPAIHR model, similar to that previously developed by Hao et al.^13^ to simulate the epidemic in Saudi Arabia’s four most afflicted regions (i.e. Eastern, Madinah, Makkah, and Riyadh). Using weekly confirmed cases, the parameters for transmission rate, documented infection rate, and effective reproduction number were estimated using the Markov Chain Monte Carlo (MCMC) approach. We modified the model to exclude inbound and outbound cases during the early stages of the Saudi Arabian pandemic given that regional travel restrictions were in place and the cancellation of important religious events (such as Hajj and Umrah). In order to model the outbreak from 13th March 2020 to 25th September 2020, we divided it into five time periods that were defined based on events and interventions: 13th March–17th April (before Ramadan), 18th April–22nd May (Ramadan and Eid), 23rd May–27th June (relaxation of some control measures), 28th June–2nd August (Hajj and Eid), and 3rd August–25th September (after most control measures had been relaxed). We made the assumption that the population of the four regions in Saudi Arabia that reported the highest number of cases would remain constant. We used the Delayed Rejection Adaptive Metropolis (DRAM) algorithm by Heikki et al.^14^ to estimate the model parameters. We fit epidemic curves to verify our parameter estimation approach and investigate the ability of our model to fit weekly incidence data.

## Methods

### Data

In this paper, we utilize data on weekly reported COVID-19 infected cases in Saudi Arabia. We analyzed the weekly infected cases from 13th March 2020 to 25th September 2020. We obtained these data from the Ministry of Health of Saudi Arabia on 25th September 2020. To maintain consistency of case definition across time periods and to distinguish between the ongoing MERS-CoV and COVID-19 outbreaks in Saudi Arabia, we only included 333732 cases with a laboratory-confirmed positive test for SARS-CoV-2. Based on this, the proposed model would be analyzed with a number of caveats in mind. First, our model depends on official data on documented cases, which will only represent a subset of the total cases. Second, regions established different testing strategies and some regions changed their approach to testing during the period analyzed. However, our model is sufficiently flexible that it is able to capture these aspects into its auto-regressive dynamics, though more detailed data could help to better understand the underlying causes behind the model estimates.

### The compartmental model, parameters, and initial conditions

We employ the SEPAIHR modelling framework presented in Fig. 1 to characterize the dynamics of COVID-19 transmission in four regions of Saudi Arabia: Eastern, Madinah, Makkah, and Riyadh.

A susceptible individual in the *S* compartment may be infected by individuals in *P*, *I*, or *A* (with different transmission rates) to move into *E* and then *P* after a latent period. At the time point of symptom onset, an individual will move from *P* to *A* or *I* based on whether they would be laboratory-confirmed in the future^13^. In order for a case to be laboratory-confirmed, the patient must be both symptomatic and have tested positive by RT-PCR, which means individuals in the *I* class must be symptomatic, while those who were in the *A* class could be asymptomatic or simply undocumented. Individuals in the *A* will eventually recover and move into the *R* class. In the meantime, individuals in the *I* class would either recover naturally and move into the *R* removal class or would develop symptoms of sufficient severity to be admitted to the hospital and therefore move into the *H* class^13^. Individuals in the *H* would eventually transition into the *R* removal class. The parameters $\gamma$ (documented infection rate) and $\beta$ (transmission rate) vary across five time periods based on events and interventions in Saudi Arabia. It is important to note that, as with all dynamic models that fit the number of documented cases at time *t*, the numbers of individuals in all compartments in this model were not directly observable except in *I*, where *I*(*t*) is the number of laboratory-confirmed cases who reported their date of symptoms onset as being at time *t*. The transmission dynamics are described by the following system of ordinary differential equations:

1 $$\begin{aligned}&\frac{dS}{dt}=-\frac{\beta S(\alpha P +\alpha A +I)}{N} \end{aligned}$$

2 $$\begin{aligned}&\frac{dE}{dt}= \frac{\beta S(\alpha P +\alpha A +I)}{N}-\frac{E}{De} \end{aligned}$$

3 $$\begin{aligned}&\frac{dP}{dt}=\frac{E}{D_e}-\frac{P}{D_p} \end{aligned}$$

4 $$\begin{aligned}&\frac{dA}{dt}=\frac{(1-\gamma )P}{D_p}-\frac{A}{D_i} \end{aligned}$$

5 $$\begin{aligned}&\frac{dI}{dt}=\frac{\gamma P}{D_p}-\frac{I}{D_i}-\frac{I}{D_q} \end{aligned}$$

6 $$\begin{aligned}&\frac{dH}{dt}=\frac{I}{D_q}-\frac{H}{D_h} \end{aligned}$$

7 $$\begin{aligned}&\frac{dR}{dt}=\frac{A+I}{D_i}+\frac{H}{D_h} \end{aligned}$$

Here $\beta$ denotes the transmission rate for documented cases, defined as the number of individuals that a documented infected case can infect per day. $\alpha$ is the ratio of the transmission rate of undocumented cases to that of documented cases. Here $\gamma$ represents the probability that a patient would be laboratory-confirmed. The latent period is defined by $D_e$ while the presymptomatic infectious period is marked by $D_p$. The symptomatic infectious period is denoted by $D_i$, the time period between the onset of illness and isolation is denoted by $D_q$, and the hospital isolation period is denoted by $D_h$. The effective reproduction number $R_t$ expressed as

8 $$\begin{aligned} R_t = \beta \alpha (\frac{1}{D_p})^{-1}+(1-\gamma )\beta \alpha (\frac{1}{D_i})^{-1}+\beta \gamma (\frac{1}{D_i}+\frac{1}{D_q})^{-1} \end{aligned}$$

where we take into account infections that occur as a result of interaction with pre-symptomatic, asymptomatic and infectious individuals.

The model parameters used for the main body of analysis are described in Table 1. We set $\alpha$ = 0.55 relying on He et al.^15^. Compartment *P* contains both documented and undocumented cases in the presymptomatic phase. We set the transmissibility of *P* to be the same as undocumented cases because it has previously been noted that the bulk of cases are undocumented^13,16^. We defined the incubation period as 5.2 days and the pre-symptomatic infectious period as $D_p$ = 2.3 days^3,15,17^. Therefore, the latent period was $D_e = 5.2 - 2.3 = 2.9$ days. As pre-symptomatic infectiousness has previously been determined to account for 44% of the total infections cases^15^, we set the mean of the total infectious period as ( $D_p$ + $D_i$) =$\frac{D_p}{0.44} = 5.2$ days, assuming constant infectiousness across the presymptomatic and symptomatic phases of documented cases^13^ thus, the mean symptomatic infectious period was $D_i$= 2.9 days. A lengthy isolation time of $D_h$ = 29 days was chosen, although this parameter had no influence on the fitting method or the final parameter estimations. The median duration from the beginning of symptoms to isolation was fixed to be $D_q$ = 10 days according to^18^.

We defined the model’s initial state based on the settings above and Table 2. The initial number of documented symptomatic cases $I\left( 0\right)$ was specified as the number of documented cases in which individuals experienced symptom onset between 2nd and 12th March 2020. We assumed the initial documented infection rate was $\gamma _0$, and thus the initial number of undocumented cases was $A(0)= \frac{1}{\gamma _0}(1-\gamma _0)I(0)$. $P_I(0)$ and $E_ I(0)$ represent the total number of documented instances in which persons developed symptoms between 13th–20th March 2020 and 15th–22nd March 2020, respectively. The initial numbers of exposed and presymptomatic individuals were then set out as follows: $E(0)=\frac{1}{\gamma _0}E_{I}(0)$ and $P(0)=\frac{1}{\gamma _0}P_{I}(0)$ respectively. We assumed $\gamma _0$ = 0.23 in our main analysis, relying on^13,19^

Taking into account the fact that the effectiveness of control measures changes with time, we assume that $\beta$ = $\beta _{1,2}$ and $\gamma$ = $\gamma _{1,2}$ for the first two periods, $\beta$ = $\beta _{3}$ and $\gamma$ = $\gamma _3$ for period 3, $\beta$ = $\beta _4$ and $\gamma$ = $\gamma _4$ for period 4, and $\beta$ = $\beta _5$ and $\gamma$= $\gamma _5$ for period 5. To estimate those parameters in each time period we assumed that the observed number of documented cases in which individuals experienced illness onset on day *d*, denoted as $y_d$, followed a Poisson distribution with rate $\lambda _d = \gamma P_{d-1} \frac{1}{D_p}$, where $P_{d-1}$ was the expected number of pre-symptomatic cases on day $(d-1)$. We fit the observed data from 13th March to 25th September (d= 1,2, ...,*D*)^20,21^. Thus, the likelihood function is;

9 $$\begin{aligned} L(\beta _1,\beta _2,\beta _3,\beta _4,\beta _5,\gamma _1,\gamma _2,\gamma _3,\gamma _4,\gamma _5) = \prod _{d=1}^{D} \frac{e^{-\lambda d}\lambda _{d}^{y_d}}{y_d !} \end{aligned}$$

We estimated these parameters by using the Monte Carlo Markov Chain method with a Delayed Rejection Adaptive Metropolis algorithm and the R package—BayesianTools (version 0.1.7)^14^. The main object in the BayesianTools package is the BayesianSetup. This class has the information about the model to be fit (likelihood), and the priors for the model parameters. A BayesianSetup is formed by the createBayesianSetup function. The function requires a log-likelihood and (optional) log-prior, and then generates the posterior and other convenience functions for samplers. In the absence of prior information, an unbounded flat prior is generated. If no explicit prior is specified, but lower and upper values are given, a standard uniform prior with the respective bounds is created, including the option to sample from this prior. We used uniform priors for $\beta _{1,5}$ following a feasibility interval to sample from *uni*(0, 2)^13,19^. Therefore, we used an informative prior of $\beta (0, 20)$ for parameters $\gamma _{1,2}$ and reparameterized $\gamma _3$, $\gamma _4$ and $\gamma _5$ by $logit (\gamma _3')= logit(\gamma _1,_2)+ \delta _3$

$$logit (\gamma _4')= logit(\gamma _3')+ \delta _4$$

$$logit (\gamma _5')= logit(\gamma _4')+ \delta _5$$

Here $logit (\gamma ')= log(\frac{\gamma }{1-\gamma })$. From the MCMC we sampled $\delta _{3,4,5}$ from the prior $N \sim (0,1)$. The results of our re-parameterization of the three parameters $\gamma _{3,4,5}$ are given by $\gamma '_{3,4,5}$. We chose a burn-in period of 40,000 iterations and ran 100,000 iterations with a sampling step size of 10 iterations. We repeated MCMC with three different sets of initial values and assessed the convergence by the trace plot and the multivariate Gelman-Rubin diagnostic using R (version 3.6.2) and R package coda (version 0.19.3)^22^. Figures S1, S2, S3, and S4 (Supplementary materials), show parameter estimates as posterior means and 95% confidence interval from 10000 MCMC samples. We utilised stochastic simulations to get the 95% credible interval of a fitted or predicted epidemic curve^20^.

As with any model of such complexity, there are many parameters that determine the dynamics. Some of these are global parameters and fit for all geographical regions (Table 1), with others used to capture the regional dynamics. Some of these parameters are fitted to the early outbreak and other data^10^. However, the others are inferred by the MCMC process. Parameters settings for our models are summarized in Table 1.

**Table 1:** Description of key model parameters not fitted in the MCMC.

Parameter	Meaning	Values	Source
$D_e$	Latent period	2.9 days	^23,24^
$D_p$	Pre-symptomatic infectious period	2.3 days	^25^
$D_i$	Symptomatic infectious period	2.9 days	Calculated
$D_q$	Duration from symptom onset to isolation	10 days	^18^
$D_h$	Isolation period	29 days	Ministry of Health in KSA
$\alpha$	Ratio of transmission rate for P and A over I	0.55	^3^

**Table 2:** Notations of compartments and the initial conditions by region for the main analysis.

Initial values	Note	Makkah	Madianh	Eastern	Riyadh	Source
S	S = N - E - P - A - I - H - R	6962521	2987366	4997867	6495550	Calculated
E	$E(0)= \frac{1}{\gamma _0}E_I(0)$, $E_I (0)$ represented the number of documented cases with symptoms starting on the day ($D_p$+1) and day($D_p + D_e$)	33417	7935	1328	2781	Calculated
P	$P(0)= \frac{1}{\gamma _0}P_I(0)$, $P_I (0)$represented the number of documented cases with symptoms starting on day 1 (13th March) and day ($D_p$)	3148	4304	694	1413	Calculated
I	*I*(0) was the number of documented cases with onset within $D_i$ days before day 1 (13th March)	205	95	51	70	MOH of KSA
A	$A(0)= \frac{1}{\gamma _0}(1-\gamma _0)I(0)$	686	281	30	176	Calculated
H	Number of isolated cases before day 1 (13th March)	23	19	30	10	MOH of KSA
R	Number of removed individuals before day 1 (13th March)	0	0	0	0	MOH of KSA
N	Total Population Size	7000000	3000000	5000000	6500000	MOH of KSA

### Prediction of the epidemic end date and resurgence risk

We conducted stochastic simulations to determine the future behaviour of the epidemic. We assume the number of individuals in each compartment in week *t* depends on the number of individuals in the previous week. We did a multinomial random sampling on a set of parameter values obtained by MCMC. Equations (1)–(7) define the SEPAIHR model, which is equivalent to the following stochastic dynamics:

10 $$\begin{aligned}&S_t - S_{t-1} = U_{S \rightarrow E} \end{aligned}$$

11 $$\begin{aligned}&E_t - E_{t-1} = U_{S \rightarrow E} - U_{E \rightarrow P} \end{aligned}$$

12 $$\begin{aligned}&P_t - P_{t-1} = U_{E \rightarrow P} - U_{P \rightarrow A} - U_{P \rightarrow I} \end{aligned}$$

13 $$\begin{aligned}&A_t - A_{t-1} = U_{P \rightarrow A} - U_{A \rightarrow R} \end{aligned}$$

14 $$\begin{aligned}&I_t - I_{t-1} = U_{P \rightarrow I} - U_{I \rightarrow H} - U_{I \rightarrow R} \end{aligned}$$

15 $$\begin{aligned}&H_t - H_{t-1} = U_{I \rightarrow H} - U_{H \rightarrow R} \end{aligned}$$

16 $$\begin{aligned}&R_t - R_{t-1} = U_{A \rightarrow R} + U_{I \rightarrow R} + U_{H \rightarrow R} \end{aligned}$$

*U* represents the number of individuals moving from one compartment to another. We predicted the week when no new documented cases would be reported and the week of clearance of all active infections in Saudi Arabian regions, assuming that the previous period’s control measures would be maintained (that is, the same parameter values). Additionally, we assessed the probability of pandemic resurgence after the relaxation of control measures. We considered easing all controls after a continual period of *t* days with no documented cases. The time to resurgence was defined as the number of days between the easing of controls and the occurrence of 100 active documented cases (*I*). We ran 10000 simulations using 10000 sets of parameter values using MCMC. Here the probability of resurgence was calculated as the proportion of simulations in which resurgence occurred.

## Results

**Figure 2:**
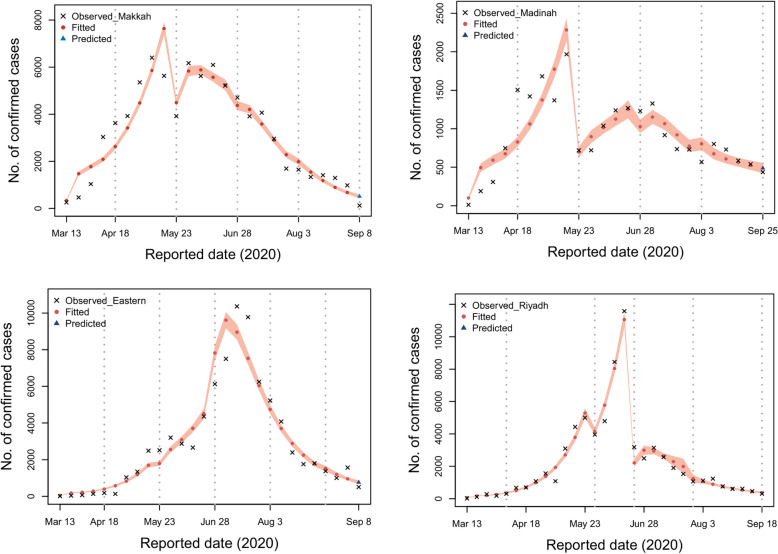
With the use of the Delay Rejection Adaptive Metropolis method, the relevant parameters were estimated for each of the four areas of interest by fitting the data from 13th March until 25th September.

Here, we examine infection dynamics in the four regions of focus to learn more about how various control interventions performed in each region. Since the first documented cases emerged in these regions, the virus was able to spread freely across much of the first and second phases with a gradual increase in the control interventions. In the four regions of Makkah, Madinah, Eastern, and Riyadh, cases peaked on 12th May (6397 cases; 95% CI 5960–9697), 15th May (1967 cases; 95% CI 1625–2308), 23rd June (10367; 95% CI 8948–11785) and 11th June (11273 cases; 95% CI 11068–12491) respectively according to the fitted model shown in Fig. 2. As the epidemic progressed, more measures were adopted to contain the disease, and the disease’s infectiousness sharply decreased after the third period. There are a few factors responsible for the sudden declining trend: first our model is dependent on official data on cases that have been documented, and these data will only ever reflect a portion of the overall number of cases. Second, different regions developed different testing strategies, and some locations altered their approach to testing during the course of the time period that was investigated. It is possible that the beginning of the Hajj term (period 4) was a contributing factor in the decrease in the number of documented cases. Additionally, previous to this time period, the government indicated that it would be increasing the size of its local testing in order to detect new cases. It is possible that the efficacy of interventions would be reduced if increases are found to be occurring during the falling phase of an epidemic. This may result in measures being kept in place for a longer period of time than they would have been had more accurate data been provided.

**Figure 3:**
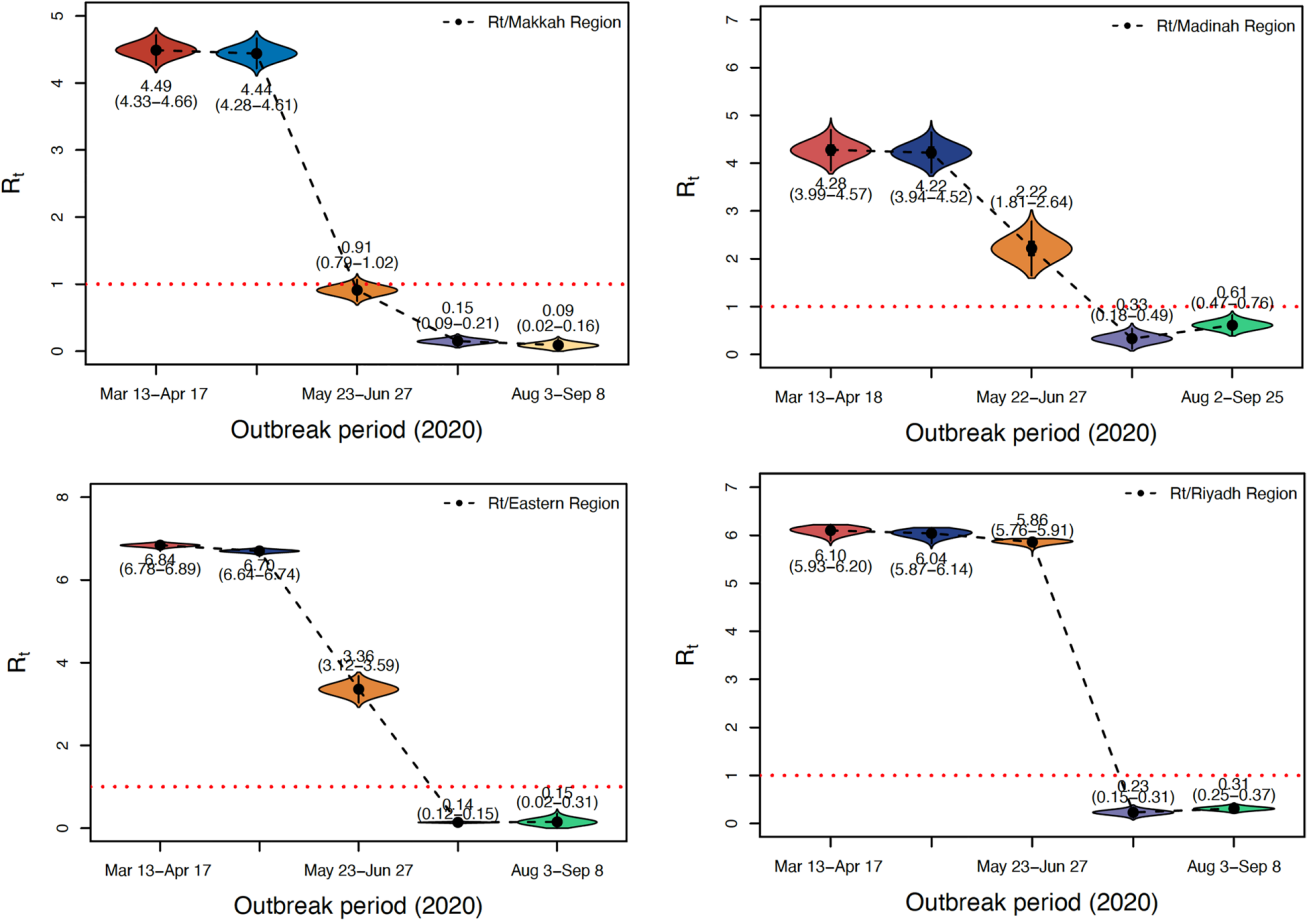
Distribution of Rt estimates derived from 10000 MCMC samples for Makkah, Madinah, Eastern, and Riyadh, respectively. The black dot in the centre of each violin plot denotes the median, the thick bar in the plot denotes the interquartile range, and the thin bar in the plot denotes the lowest and maximum values. The mean and the credible interval for 95%, which is shown in parentheses, are labelled below or above, respectively.

We estimate the effective reproduction number $R_t$ as an indicator of SARS-CoV-2 transmission before and after the interventions. Figure 3 depicts the dramatic shift in the rate of SARS-CoV-2 transmission as a result of decreased social contact and other control measures. At the beginning of the pandemic, $R_t$ for SARS-CoV-2 in Saudi regions was between 4 and 6 as illustrated in Tables 6 and 7. In other words, on average each case spread to between four and six others. Considering that each new generation of SARS-CoV-2 cases occurs every five days, it is evident that this pandemic was rapidly expanding out of control. Moreover, we assumed that the transmission rate and the documented infection rate did not change during the first two periods since interventions were carried out gradually until a complete lockdown took place. As more measures were introduced, the spread of the disease began to decrease. Therefore, our data were based on the weekly reported number of documented SARS-CoV-2 cases broken down by region. As a result, it became clear that the reliability of the $R_t$ value was relatively high for transmission.

**Figure 4:**
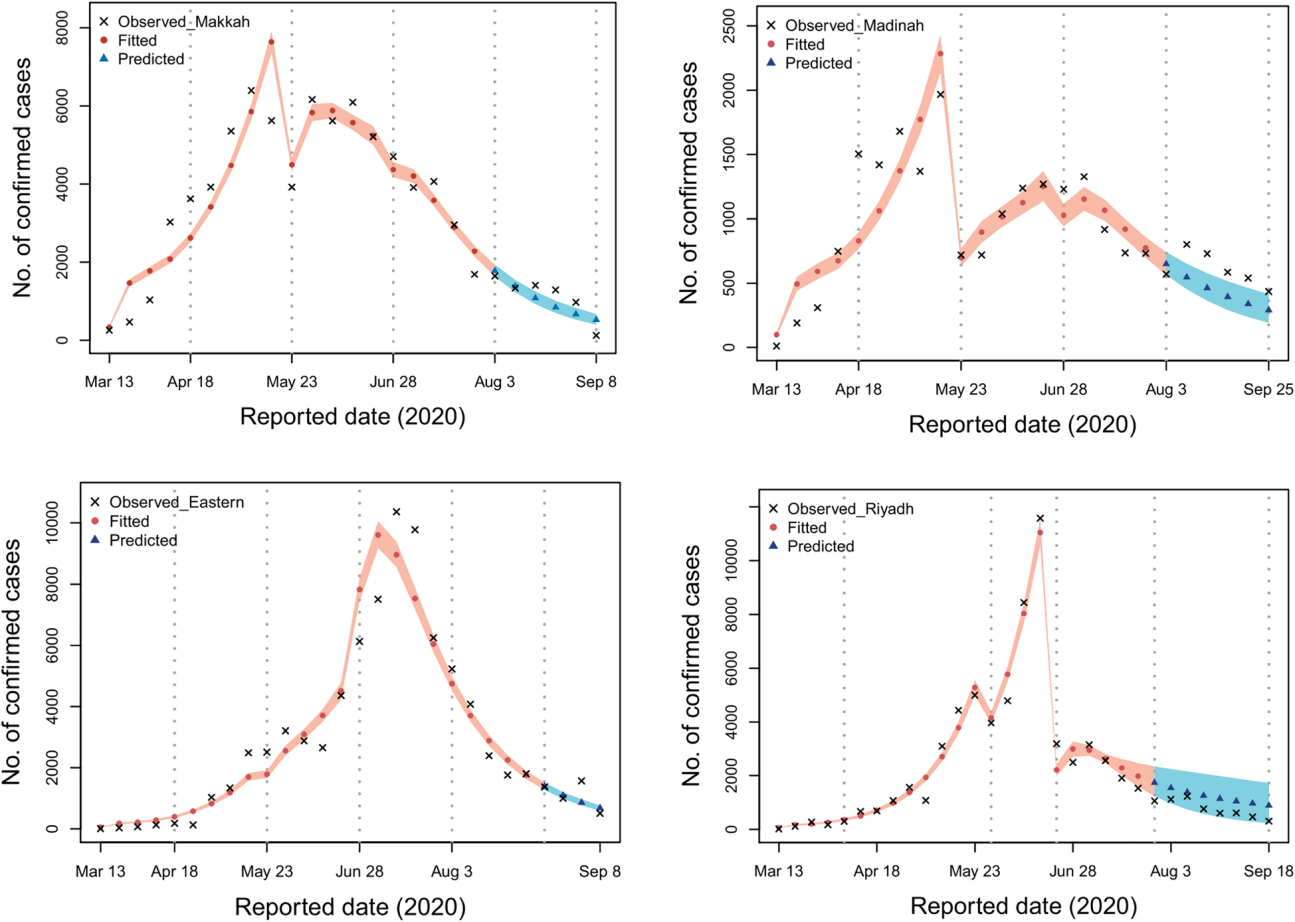
The relevant parameters were estimated for each of the four regions of interest by first fitting the data of each region, and then predicting using the parameters from period 4. This was done with each of the four regions of interest separately.

The effects of the events and interventions on the dynamics of SARS-CoV-2 in the regions of interest are considered. First, if the controls remained in phase four in Makkah, our model projects that the total number of documented cases would increase to 81047 (95% CI 79421–82672). In Al-Madinah, the cumulative number of documented cases would have increased to 22997 (95% CI 19578–26415). The number of cumulative documented cases may have reached 80520 (95% CI 78335–82704) if controls stayed steady in the Eastern region at the level they were at in phase four. If the pattern shown during the fourth period is taken into account, we estimate that there would have been 67150 (95% CI 63731–70568) documented infections in the Riyadh region. Figure 4 illustrates these findings.

**Figure 5:**
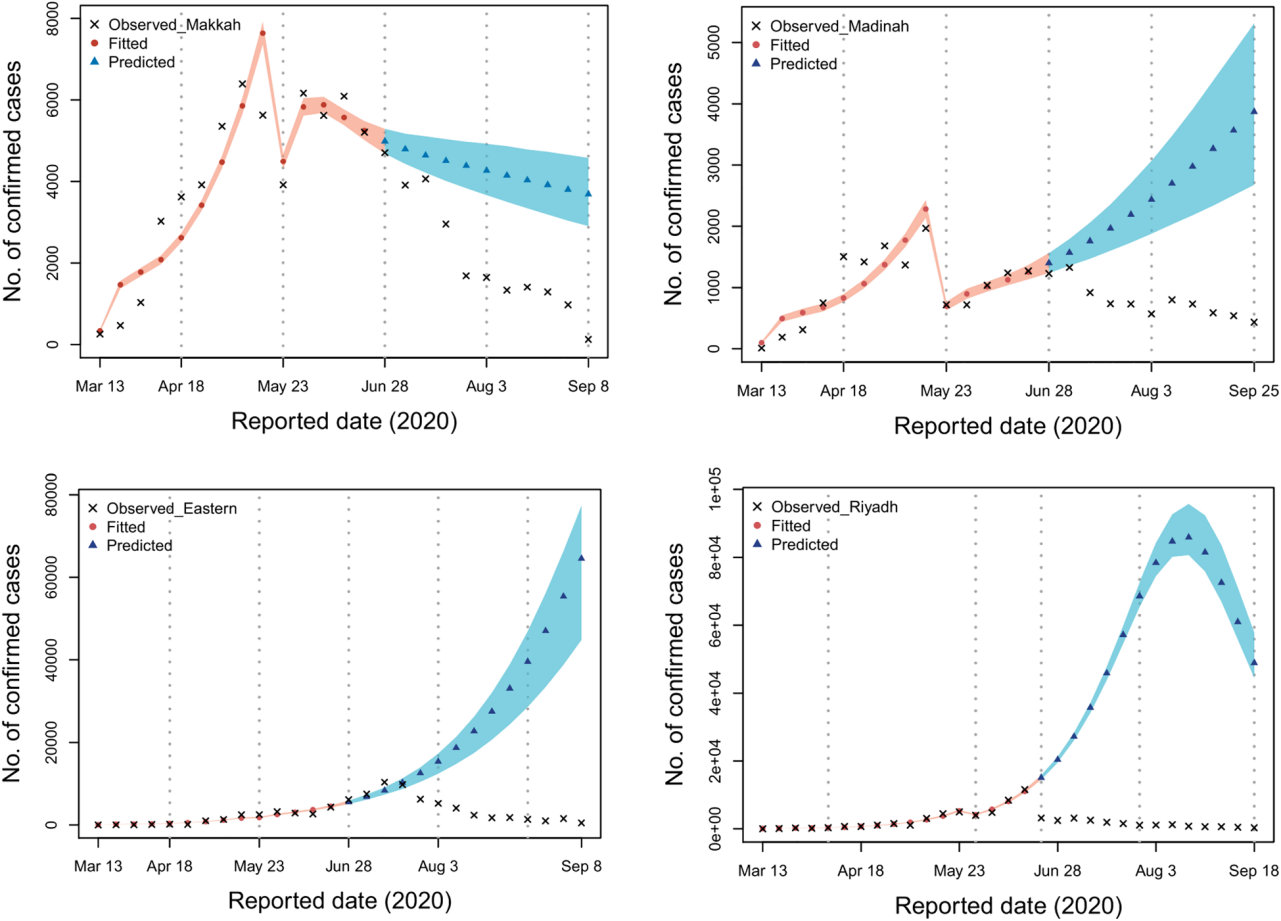
For each of the four areas of interest, the relevant parameters were estimated by first fitting the data of each region and then predicting using the parameters from period 3. This was carried out for each of the four areas of interest independently.

We now explore the impact of controls remaining in place at the same level as that implemented in phase three. In that case, the number of documented cases in Makkah would have increased to 116641 (95% CI 105015–128266). Similarly, the total number of documented cases in Al-Madinah would have increased to 53877 (95% CI 50458-57295) if the outbreak had been allowed to continue at the same level. If the controls had remained unchanged from how they were in phase three in the Eastern region, the total number of documented cases would have been 310459 (95% CI 298362–334981). Finally, in Riyadh this would have resulted in 665241 documented cases (95% CI 651822 to 678659). Figure 5 highlights these findings.

**Figure 6:**
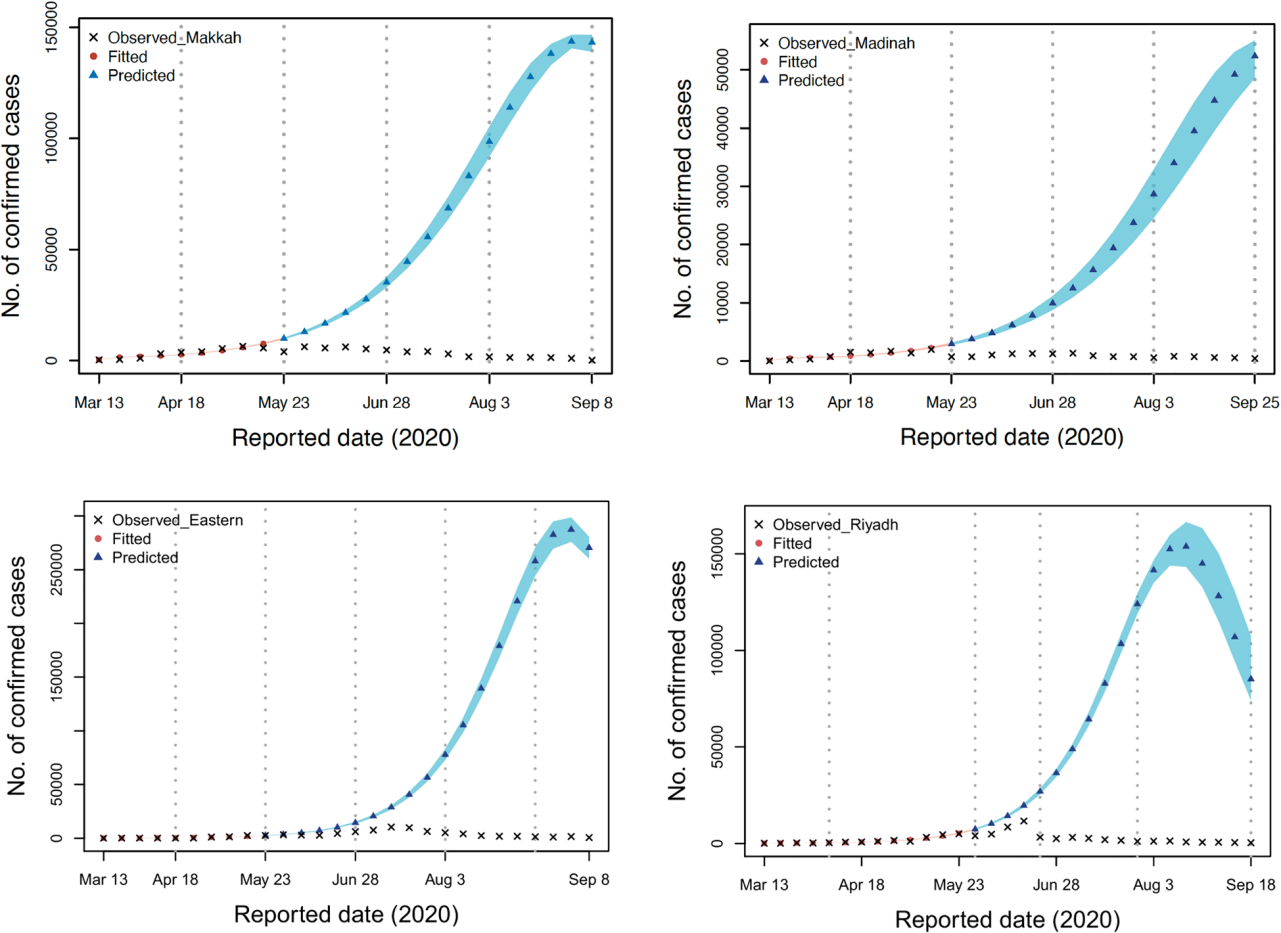
For each of the four regions of interest, the relevant variables were identified by first fitting the data from each area and then making predictions using the parameters from period 2. Each of the four regions of interest was done separately.

We now investigate the impact of second-period controls remaining in place. In that case, the number of documented cases would increase to 1236642 (95% 1218314–1251626), 442865 (95% CI 439446–456283), 454031 (95% CI 441846–466215), and 2322624 (95% CI 1919206-3026042) in the regions of Makkah, Madinah, Eastern, and Riyadh, respectively (see Fig. 6).

The efficacy of NPIs is dependent on when they are adopted, with earlier adoption resulting in greater success in lowering transmission rates of infectious diseases. In the early stages of COVID-19, Saudi regions made the decision to gradually implement measures in order to understand the severity of the disease and reduce the economic and social costs of lockdowns, as well as the political costs. In Fig. 6, if the government were to rely on the interventions of the second phase, then the number of cases of infection would considerably rise owing to the ineffectiveness of the measures. In the third period as in Fig. 5, the government made it possible to relax some of the control measures, but it is ultimately up to each area to decide whether they will maintain the same level of control or whether they will increase or decrease it. In comparison to the control measures carried out during the third and fourth periods, this led to significantly improved outcomes. The reason that these time periods were chosen is that there was no stiffening of the NPI response in most Saudi regions during the first two periods and control interventions were improved later on.

**Figure 7:**
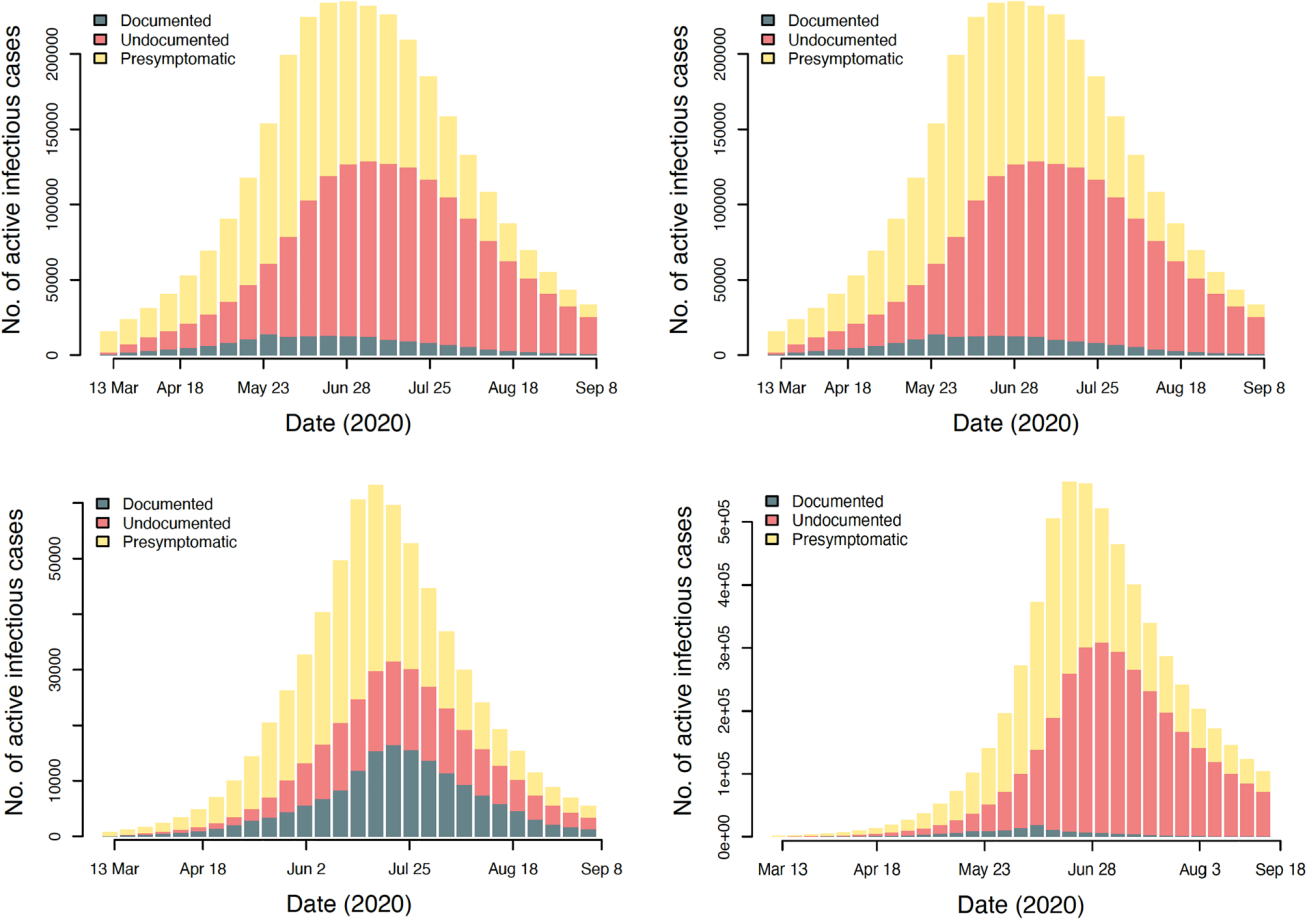
The estimated number of infected cases that were active (presymptomatic, symptomatic, and asymptomatic) during the research period in the areas of Makkah, Madinah, Eastern, and Riyadh respectively.

Significant undetected infections resulted in the fast spread of new coronaviruses (SARS-CoV-2) which is illustrated in Fig. 7. The proportion of undocumented infections, including asymptomatic cases and undocumented symptomatic individuals who did not seek medical treatment or be tested for mild symptoms, was greater than that of Wuhan at the onset of the pandemic^26^, which may be a result of the following factors: first, the medical configuration was not optimal and public awareness was limited during the onset of the pandemic while the undocumented rate progressively increased; Second, contact tracing procedures employed in Saudi regions may have become overwhelmed if the number of early-stage cases in Saudi regions rises substantially. The discrepancy between the predicted proportions of asymptomatic (undocumented) cases may be attributable to the difficulty in the un-identifiability of parameters in epidemiological models. There were a substantial number of asymptomatic infected individuals with high infectivity in Saudi regions, where the epidemic situation escalated rapidly. Our research emphasises the frequency of asymptomatic SARS-CoV-2 cases and their role in transmission in order to increase people’s knowledge of asymptomatic cases and to serve as a guide for the prevention and control of SARS-CoV-2.

**Table 3:** Estimated transmission rate in Saudi Regions.

Region	$\beta _1$	$\beta _2$	$\beta _3$	$\beta _4$	$\beta _5$
Eastern	1.99 (1.98–2.0)	1.99 (1.98–2.0)	1.07 (0.99–1.15)	0.04 (0.04–0.05)	0.05 (0.01–0.11)
Madinah	1.64 (1.52–1.76)	1.64 (1.52–1.76)	0.94 (0.77–1.12)	0.14 (0.08–0.21)	0.27 (0.2–0.34)
Makkah	1.73 (1.66–1.8)	1.73 (1.66–1.8)	0.38 (0.34–0.43)	0.06 (0.04–0.09)	0.04 (0.01–0.07)
Riyadh	1.96 (1.9–2.0)	1.96 (1.9–2.0)	1.98 (1.95–2.0)	0.08 (0.05–0.11)	0.11 (0.09–0.13)

**Table 4:** Estimated ascertainable infection rate.

Region	$\gamma _1$	$\gamma _2$	$\gamma '_3$	$\gamma '_4$	$\gamma '_5$
Eastern	0.6 (0.58–0.62)	0.6 (0.58–0.62)	0.44 (0.42–0.46)	0.61 (0.57–0.67)	0.67 (0.61–0.76)
Madinah	0.26 (0.24–0.28 )	0.26 (0.24–0.28)	0.06 (0.05–0.07)	0.04 (0.04–0.06 )	0.05 (0.04–0.0)
Makkah	0.25 (0.24–0.26)	0.25 (0.24–0.26)	0.11 (0.1–0.12)	0.09 (0.09–0.11)	0.10 (0.09–0.13)
Riyadh	0.26 (0.25–0.28)	0.26 (0.25–0.28)	0.14 (0.14–0.16)	0.02 (0.02–0.02)	0.022 (0.02–0.03)

In this model, we fitted dynamic transmission rates because of varied preventable measures by the Saudi government at the level of the country or region. After a series of actions taken by the government, regions and cities went into lockdown, resulting in a decrease in the transmission rate as in Table 3. Before the interventions were introduced, in the first two periods of our study, we assumed the transmission rate did not change since individual and community responses had not effectively taken place. After severe interventions were implemented, the transmission rates were allowed to vary in later periods and reduced gradually due to the control measures that reduced the spread of disease^27^. Estimates of documented infection rates are presented in Table 4. Our model estimates show the documented infection rate has continued to decrease in the last two periods. Thus, the parameters we fit across periods are a measure of how effective the lockdown was in bringing down the documented infection rate^28^.

### Risk of resurgence

The risk of resurgence in Saudi Arabia’s four regions has been examined in this section after the relaxation of intervention measures. There will be a rise in disease activity if control measures are relaxed without taking into account increases in the number of cases being detected, isolated, and/or traced. We predict the first week of no new cases of infection and the week when all current infections in Saudi Arabia will be eradicated.

In the Makkah region, had the trend continued into the fourth period, the number of documented infections would have dropped to zero on average by the 6th September (23rd August to 27th September), and all infections would have been eradicated by the 26th of October (7th October to 14th November). On the 28th June, the number of weekly active infections (including presymptomatic, symptomatic, and asymptomatic cases) reached its highest point of 230,230 (95% CI 226811–234364), and on 8th September, that number dropped to 44023 (95% CI 40604–47441).

Therefore, the number of documented infections would have reached zero in Al-Madinah region on average on 6th November (23rd October to 22nd November), and all infections would have been eliminated by 1st December (27th November to 14th December). On 23rd June, weekly active infections (including presymptomatic, symptomatic, and asymptomatic cases) peaked at 130,134 (95% CI 126715–133552) and then declined to 60023 (95% CI 58604–63441) on 25th September.

If the trend had continued as it did in the fourth period in the Eastern region, the average number of documented infections would have reached zero on 2nd November (from 23rd October to 18th November), and the total eradication of infections would have happened on 1st December (26th November to 22nd December). The number of weekly active infections (including presymptomatic, symptomatic, and asymptomatic cases) peaked at 65000 (95% CI 61581–68418) during the week of July 23rd and subsequently decreased to 800 (95% CI 765–834) on 8th of September.

Lastly, the model predicted that the number of weekly active infections in the Riyadh region (including presymptomatic, symptomatic, and asymptomatic infections) peaked on 28th June at 562332 (95% CI 513379–619542) and then decreased to 188215 (95% CI 174796–191633) on 18th September. On average, we expected that the number of documented infections would have decreased to zero on 18th October (7th October to 14th November) and that the total number of infections would have been eliminated on 1st December if the trend continued as it did in the fourth period (20th November to 23rd December). Figure 7 illustrates these findings. We found that if control measures were lifted 30 days following the first day of zero documented cases.

**Figure 8:**
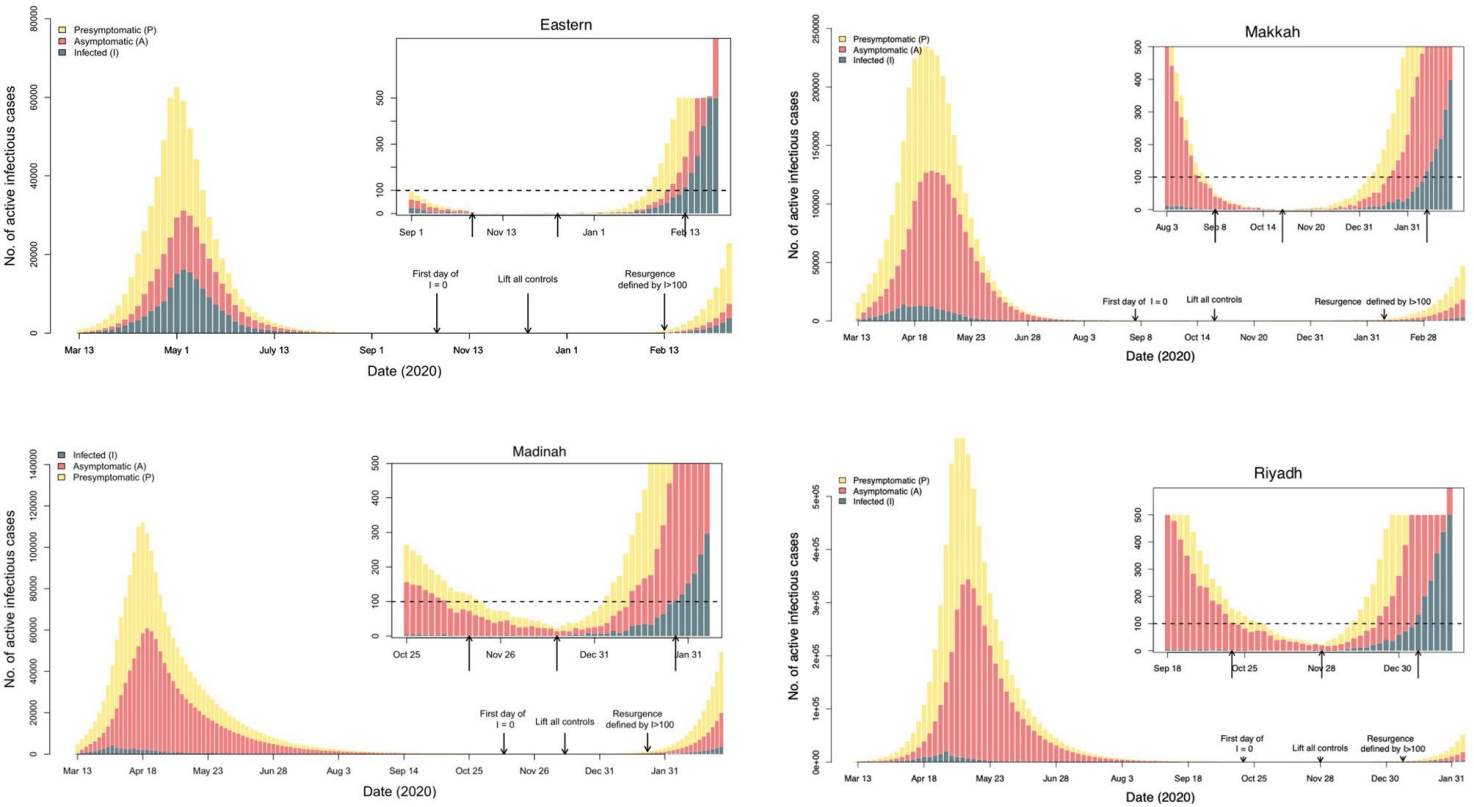
Figure demonstrating the effect of relaxing all control measures in all four regions 30 days following the first day without confirmed cases.

The probability of resurgence, which we define as the number of active documented cases greater than 100 could be as high as 0.96 in Eastern, 0.95 in Madinah, 0.97 in Makkah, and 0.96 in Riyadh. If we adopt more stringent conditions of lifting controls after observing no confirmed cases for a continuous period of 30 days, the probability of resurgence decreases to 0.31, 0.28, 0.30, and 0.30, with probable resurgence occurring on 13th February, 7th February, 2nd January, and 8th January for Eastern, Makkah, Madinah and Riyadh, respectively (Fig. 8). Despite the use of a simplified model, these results emphasize the hazards of ignoring undetermined occurrences when modifying intervention techniques.

### Sensitivity analysis

For the purpose of testing the robustness of our research results, we conducted a series of sensitivity analyses by varying the durations of the latent and infectious periods, the ratio of transmissibility in asymptomatic (undocumented) cases to symptomatic (documented) cases, and the initial documented infection rate. We conduct eight sensitivity analyses (S1 to S8) within each model for each region of Saudi Arabia to assess the robustness of our model results. For instance, the sensitivity analysis performed for S1 was based on the changes of the latent period and pre-symptomatic infectious period, respectively, and other parameters remain the same. These modifications were carried out with the help of reference^15,29^, and the same approaches were used for the other parts of the sensitivity analysis, which is summarised in Table 5.

**Table 5:** Description of essential model parameters that were not fitted in the MCMC, where $D_e$ refers to the latent period, $D_p$ refers to the pre-symptomatic infectious period, $D_i$ refers to the symptomatic infectious period, $\gamma _0$ refers to the initial ascertain rate and $\alpha$ refers to the ratio of the transmission rate for *P* and *A* to *I*.

Parameter	Main Analysis	S1	S2	S3	S4	S5	S6	S7	S8	Source
$D_e$	2.9	4	2.9	2.9	2.9	2.9	3	2.9	2.9	^15,29^
$D_p$	2.3	3	2.3	2.3	2.3	2.3	1.1	2.3	2.3	^29^
$D_i$	2.9	2.9	2.9	2.9	2.9	2.9	2.9	2.9	4.6	^30^
$\gamma _0$	0.23	0.23	0.23	0.23	0.14	0.42	0.23	0.23	0.10	^13,21^
$\alpha$	0.55	0.55	0.46	0.62	0.55	0.55	0.55	0.50	0.55	^30,31^

In particular, for (S1), we raised the incubation period to 7 days (upper 95% CI based on ref^15^) and the pre-symptomatic infectious period to 3 days (upper 95% CI based on ref^29^). Therefore we set $D_e = 4$ and $D_p=3$, and modified $E_0$ and $P_0$ as needed. The transmissibility of the undocumented cases was assumed to be 0.46 (lower 95 % CI according to ref.^31^) of the infection cases for (S2); for (S3), the transmissibility of the asymptomatic (undocumented) cases was assumed to be 0.62 (upper 95 % CI according to ref^31^). We assumed that in (S4), the initial documented infection rate was $\gamma _0$ = 0.14 (lower 95 % CI according to ref^13^) and adjusted $A_0$, $P_0$ and *E*(0) accordingly. Similarly for (S5) we assumed the initial documented infection rate was $\gamma _0$ = 0.42 (upper 95 % CI according to ref^13^) and adjusted $P_0$, $A_0$, and $E_0$ accordingly. In (S6) we set the variables $D_ e=3$ and $D_p=1.1$, and altered the values of $P_0$ and $E_ 0$ as necessary in accordance with^13^. In (S7) we assumed that the transmission rate of asymptomatic (undocumented) cases was half that of documented cases by setting 0.5. Finally, in (S8) we assumed that the infectious period $(D_i)$ was double that of symptomatic cases by setting 6 days. Both (S7) and (S8) were based on^30^. The results of our sensitivity analysis are summarised in Tables 6 and 7. We note that the variation in the model predictions of $R_t$ varies from setting to setting. However, these variations appear to be fairly small, proposing the robustness of the results to the specification of associated values in fairly realistic ranges^13,13^. Our sensitivity analysis provides information about the importance of each parameter to the model representing the transmission of SARS-CoV-2. An increase (or decrease) in parameter values, while other parameters’ values remain the same, contributes to an increase (or decrease) in effective reproduction numbers. For example, an increase in infectious period would result in a higher effective reproduction number at the beginning of the epidemic and a longer time required to clear all infections in Saudi regions^32^. Our sensitivity analysis indicates that almost all model parameters may have an important role in spreading this virus among susceptible people. In particular, the contact rate from person-to-person and the transition rate of asymptomatic (undetected) individuals play a significant role in disease spread. Our important findings, of a significant decrease in $R_t$ after interventions and the existence of a substantial number of presymptomatic and asymptomatic cases, were found to be robust. This highlights that Saudi authorities should pay attention to intervention strategies in the event of a resurgence of cases and quarantining those who were in contact with active cases can effectively reduce the disease^33^. In Tables 6 and 7 we show the estimated effective reproduction number $R_t$ associated with 95% CIs obtained from those eight sensitivity analyses for all four regions and all five time periods.

**Table 6:** Sensitivity analysis of the effective reproduction number $R_t$ for Eastern and Madinah.

Analysis	Region	13th Mar–17th Apr	18th Apr–22nd May	23rd May–27th Jun	28th Jun–3rd Aug	4th Aug–25th Sep
Main	Eastern	6.84 (6.78–6.89)	6.7 (6.64–6.74)	3.34 (2.91–3.60)	0.14 (0.12–0.16)	0.15 (0.02–0.31)
S1	Eastern	7.06 (7.02–7.09)	6.99 (6.94–7.02)	2.56 (2.33–2.83)	0 (0–0)	0.11 (0.01–0.34)
S2	Eastern	6.4 (6.34–6.46)	6.24 (6.19–6.3)	3.32 (3.1–3.57)	0.14 (0.12–0.15)	0.15 (0.02–0.29)
S3	Eastern	7.24 (7.19–7.28)	7.11 (7.06–7.15)	3.39 (3.13–3.65)	0.14 (0.12–0.16)	0.14 (0.02–0.29)
S4	Eastern	6.43 (6.39–6.47)	6.34 (6.3–6.37)	3.4 (3.17–3.67)	0.14 (0.13–0.16)	0.14 (0.03–0.28)
S5	Eastern	7.08 (7.02–7.13)	6.91 (6.86–6.96)	3.33 (3.1–3.56)	0.14 (0.12–0.15)	0.15 (0.03–0.3)
S6	Eastern	6.93 (6.88–6.97)	6.82 (6.77–6.86)	3.48 (3.22–3.74)	0.06 (0.05–0.08)	0.19 (0.04–0.36)
S7	Eastern	6.23 (6.18–6.26)	6.16 (6.12–6.2)	3.36 (2.99–3.67)	0.15 (0.13–0.18)	0.16 (0.02–0.45)
S8	Eastern	6.97 (6.92–7.02)	6.82 (6.77–6.87)	3.39 (3.16–3.64)	0.14 (0.12–0.15)	0.14 (0.02–0.28)
Main	Madinah	5.14 (4.73–5.51)	5.1 (4.7–5.47)	1.71 (1.14–2.29)	0.18 (0.02–0.4)	0.61 (0.36–0.86)
S1	Madinah	5.15 (4.75–5.51)	5.11 (4.71–5.47)	1.71 (1.16–2.32)	0.19 (0.02–0.41)	0.6 (0.37–0.85)
S2	Madinah	4.28 (3.99–4.57)	4.23 (3.94–4.52)	2.22 (1.84–2.63)	0.34 (0.20–0.50)	0.61 (0.47–0.76)
S3	Madinah	4.17 (3.9–4.47)	4.14 (3.86–4.43)	2.23 (1.82–2.66)	0.34 (0.19–0.5)	0.61 (0.46–0.76)
S4	Madinah	4.25 (3.96–4.54)	4.22 (3.93–4.5)	2.34 (1.91–2.78)	0.36 (0.2–0.53)	0.66 (0.51–0.82)
S5	Madinah	4.43 (4.12–4.72)	4.35 (4.04–4.64)	2.11 (1.73–2.52)	0.32 (0.18–0.47)	0.58 (0.45–0.72)
S6	Madinah	4.17 (3.93–4.42)	4.08 (3.84–4.33)	2.52 (2.22–2.85)	0.48 (0.36–0.6)	0.58 (0.47–0.69)
S7	Madinah	4.23 (3.95–4.54)	4.18 (3.91–4.49)	2.23 (1.81–2.67)	0.34 (0.19–0.5)	0.61 (0.47–0.76)
S8	Madinah	4.58 (3.12–5.04)	4.54 (3.08–5)	2.18 (1.56–3.7)	0.16 (0.01–0.37)	0.51 (0.1–0.8)

**Table 7:** Sensitivity analysis of estimated effective reproduction number $R_t$ for Makkah and Riyadh.

Analysis	Region	13th Mar–17th Apr	18th Apr–22nd May	23rd May–27th Jun	28th Jun–3rd Aug	4th Aug–25th Sep
Main	Makkah	4.49 (4.33–4.66)	4.44 (4.28–4.61)	0.91 (0.79–1.02)	0.15 (0.09–0.21)	0.09 (0.02–0.15)
S1	Makkah	5.53 (5.34–5.63)	5.49 (5.3–5.59)	0.27 (0.11–0.7)	0.04 (0–0.23)	0.01 (0–0.05)
S2	Makkah	4.48 (4.3–4.65)	4.43 (4.26–4.6)	0.91 (0.79–1.02)	0.15 (0.09–0.21)	0.09 (0.03–0.15)
S3	Makkah	4.39 (4.23–4.55)	4.35 (4.2–4.52)	0.91 (0.8–1.03)	0.15 (0.09–0.21)	0.09 (0.02–0.16)
S4	Makkah	4.48 (4.32–4.65)	4.45 (4.29–4.62)	0.96 (0.83–1.09)	0.16 (0.09–0.22)	0.09 (0.02–0.16)
S5	Makkah	4.59 (4.42–4.75)	4.5 (4.33–4.66)	0.86 (0.76–0.98)	0.14 (0.08–0.2)	0.09 (0.03–0.15)
S6	Makkah	4.27 (4.24–4.29)	4.2 (4.16–4.22)	1.39 (1.3–1.48)	0.23 (0.18–0.28)	0.17 (0.13–0.22)
S7	Makkah	4.45 (4.28–4.62)	4.4 (4.24–4.57)	0.91 (0.8–1.03)	0.15 (0.09–0.21)	0.09 (0.02–0.16)
S8	Makkah	4.56 (4.39–4.73)	4.51 (4.34–4.68)	0.91 (0.8–1.03)	0.15 (0.09–0.21)	0.08 (0.02–0.15)
Main	Riyadh	6.10 (5.88–6.20)	6.10 (5.82–6.14)	5.88 (5.78–5.93)	0.57 (0.16–1.06)	0.33 (0.25–0.43)
S1	Riyadh	6.83 (6.79–6.85)	6.79 (6.75–6.81)	6.56 (6.25–6.65)	0.04 (0–0.17)	0.2 (0.11–0.29)
S2	Riyadh	5.57 (5.52–5.59)	5.49 (5.44–5.51)	5.14 (5.08–5.17)	0.22 (0.15–0.3)	0.29 (0.23–0.35)
S3	Riyadh	6.19 (5.96–6.43)	6.14 (5.91–6.38)	6.47 (6.28–6.56)	0.23 (0.14–0.32)	0.33 (0.26–0.39)
S4	Riyadh	6.29 (6.09–6.49)	6.19 (5.98–6.39)	5.97 (5.81–6.06)	0.2 (0.13–0.28)	0.29 (0.23–0.34)
S5	Riyadh	6.16 (5.98–6.29)	6.09 (5.91–6.22)	5.89 (5.77–5.95)	0.22 (0.14–0.3)	0.3 (0.25–0.36)
S6	Riyadh	5.04 (4.94–5.09)	4.95 (4.85–5)	4.67 (4.62–4.69)	0.59 (0.52–0.66)	0.34 (0.29–0.39)
S7	Riyadh	5.81 (5.74–5.85)	5.74 (5.67–5.78)	5.45 (5.37–5.48)	0.23 (0.15–0.31)	0.3 (0.24–0.35)
S8	Riyadh	5.89 (5.79–5.94)	5.86 (5.76–5.92)	5.78 (5.72–5.81)	0.3 (0.21–0.41)	0.38 (0.31–0.46)

## Discussion

In this paper, we give estimates of the epidemiological parameters of the SARS-CoV-2 outbreak in Saudi Arabian regions, as well as predictions based on NPIs, particularly about the proportion of documented cases when the lockdown policy is lifted^19^. Prior to the shutdown, estimates of effective reproductive ratios for Saudi Arabian regions varied between 4.28 (95% CI 3.99–4.57) and 6.84 (95% CI 6.78–6.89), but because of the uncertainty surrounding these estimates, they are not significantly different across regions. Thus, observed variations in case numbers were attributed to the pandemic beginning in the Eastern and Makkah regions initially^34^. The model estimated the effect of the lockout on the effective reproductive ratio, assuming a significant fall in $R_t$ after the lockdown. Additionally, the method simulates the population size of those who have been or were actively infected as of 25th September 2020.

Our findings must be interpreted in light of the mechanistic model shown in Fig. 1, and special attention must be paid to the parameters extracted from the scientific literature and documented in Table 2 since new estimates are released daily. To begin, our model considers the estimate of transmission rate and three distinct types of infectious cases: documented cases *I*, asymptomatic (undocumented) *A* cases, and presymptomatic *P* cases. *I* is the number of documented infected individuals verified by a positive PCR SARS-CoV-2 test in our observation model. Thus, *P* and *A* might be viewed as undocumented symptomatic instances that can be documented during a routine visit to the general practitioner (possibly through remote teleconsultation). This is a highly simplified picture of the SARS-CoV-2 infection, which may manifest itself in a variety of ways (e.g. asymptomatic, moderate, severe), each of which can be depicted as a separate compartment. Our model does not include a compartment for SARS-CoV-2 patients in the ICU, nor does it include an inflow of susceptibles (and corresponding outflow) from other regions. However, population movements across regions were limited throughout the lockdown phase. In the *R* compartment, deaths were not separated from recoveries, although this had no effect on the primary estimations during the course of the study’s observation period^26^. Our model is based on a statistical study of critical parameters deduced from available data. As a consequence, the more data that are available, the more precise the parameter estimates will be.

Our findings show that the non-pharmaceutical interventions taken by the Saudi national and regional governments had a significant effect on reducing spread, albeit with some regional variations^35^. We observed an early deceleration in the spread of the epidemic in all regions, due to the introduction of non-pharmaceutical intervention measures that preceded the lockdown^36^. After that, the nationwide lockdown further reduced the value of $R_t$ in all Saudi regions. A full lifting of intervention measures, in the absence of vaccination, would likely have led to a significant resurgence in cases in Saudi Arabia. In the longer term, in order for measures to be lifted, it is clear that a large-scale vaccination program would be required, along with targeted testing and tracing of individuals and potentially local control measures in order to minimize the potential for a rise in cases in the future. Such strategies have been adopted in other countries, with successful vaccination campaigns across several countries, particularly in Europe and the USA^37^ and a similar approach may allow us to achieve the same in Saudi Arabia^38^.

Some limitations of our study should be noted. Firstly, not all cases are reported and there is a delay in confirmation—we may therefore have underestimated the overall documented infection rate in this study. Secondly, we ignored clinically diagnosed cases without laboratory confirmation, which would lead to lower estimates of documented infection rates. Thirdly, the primary objective of this research is not to produce detailed projections of SARS-CoV-2 cases in various age and other demographic groups. Due to the limited number of COVID-19 cases in Saudi Arabia, it is challenging to assess contact rates across finely stratified age groups. Therefore, stratifying the model at this level would create an excessive number of unknown parameters. Instead, we aim to construct a coarse-grained model that can assess control interventions that are differently influenced by events and interventions in Saudi Arabia and may assist in identifying potential risks in specific places. Future work that models heterogeneous transmission between different groups with data from other regions will lead to deeper insights into the effectiveness of different control strategies that may inform the authorities in Saudi Arabia regarding how restrictions may be lifted in the future.

In conclusion, the implementation and optimal timing of NPIs is determined by an analysis of the possible costs and benefits, from a health, economic, and social perspective, of various policy choices. However, pandemics like that caused by SARS-CoV-2 make the deployment of such controls a worldwide public benefit that has to be prioritized. It is essential to investigate the challenges that nations must overcome and determine how resources might be more effectively distributed in order to enhance the timely implementation of these actions. We show evidence in the case of Saudi Arabia that these lockdowns, particularly those accompanied by curfews and cancellation of religious gatherings, or harsher stay-at-home orders that restricted physical mobility, had a significant effect upon reducing transmission. A complete and immediate release of lockdown, as evidenced in other published models in other settings^39,40^, would result in a resurgence of infection. Additional measures, such as strict case isolation and contact tracing^41^, may be effective in reducing the number of new infections. Research in the future should investigate the financial and societal costs of these preventative measures, as well as ways in which various iterations of these preventative measures might optimize the decrease in disease transmission while reducing expenses.

## Supplementary Information

Supplementary Information.

## Data Availability

The datasets generated and/or analysed during the current study are available in the Ministry of Health of Saudi Arabia repository, https://www.moh.gov.sa/en/Ministry/OpenData/Pages/default.aspx.

## References

[1] Yang C, Wang J (2020). A mathematical model for the novel coronavirus epidemic in Wuhan, China. Math. Biosci. Eng..

[2] She J, Jiang J, Ye L, Lijuan H, Bai C, Song Y (2020). 2019 novel coronavirus of pneumonia in Wuhan, China: emerging attack and management strategies. Clin. Transl. Med..

[3] Li, Q., *et al.* Early transmission dynamics in Wuhan, China, of novel coronavirus–infected pneumonia. *N. Engl. J. Med.* (2020). 10.1056/NEJMoa2001316 PMC7121484 31995857

[4] Zhou, P., *et al*. Discovery of a novel coronavirus associated with the recent pneumonia outbreak in humans and its potential bat origin. BioRxiv (2020).

[5] Mourier T, Shuaib M, Hala S, Mfarrej S, Alofi F, Naeem R, Alsomali A, Jorgensen D, Subudhi AK, Rached FB (2022). SARS-CoV-2 genomes from Saudi Arabia implicate nucleocapsid mutations in host response and increased viral load. Nat. Commun..

[6] Salih, H.M.A., Ahmed, S.O., Yara, A.N., *et al.* Coronavirus disease 2019 (covid-19): Emerging and future challenges for gulf states. Authorea Preprints (2020).

[7] Ahmad N (2020). Covid-19 modeling in Saudi Arabia using the modified susceptible-exposed-infectious-recovered (SEIR) model. Cureus.

[8] Al-Hadeethi Y, El Ramley IF, Sayyed MI (2021). Convolution model for covid-19 rate predictions and health effort levels computation for Saudi Arabia, France, and Canada. Sci. Rep..

[9] Amer F, Hammoud S, Farran B, Boncz I, Endrei D (2021). Assessment of countries’ preparedness and lockdown effectiveness in fighting covid-19. Disaster Med. Public Health Prep..

[10] Keeling, M. J., *et al*. Fitting to the UK covid-19 outbreak, short-term forecasts and estimating the reproductive number. Stat. Methods Med. Res. 09622802211070257 (2020). 10.1177/09622802211070257 PMC9465059 35037796

[11] Imai, N., Cori, A., Dorigatti, I., Baguelin, M., Donnelly, C.A., Riley, S., & Ferguson, N. M. Report 3: transmissibility of 2019-ncov. Imperial College London (2020).

[12] Tang Y, Serdan TDA, Alecrim AL, Souza DR, Nacano BRM, Silva FLR, Silva EB, Poma SO, Gennari-Felipe M, Iser-Bem PN (2021). A simple mathematical model for the evaluation of the long first wave of the covid-19 pandemic in brazil. Sci. Rep..

[13] Hao X, Cheng S, Degang W, Tangchun W, Lin X, Wang C (2020). Reconstruction of the full transmission dynamics of covid-19 in Wuhan. Nature.

[14] Haario H, Laine M, Mira A, Saksman E (2006). Dram: Efficient adaptive MCMC. Stat. Comput..

[15] He X, Lau EHY, Wu P, Deng X, Wang J, Hao X, Lau YC, Wong JJ, Guan Y, Tan X (2020). Temporal dynamics in viral shedding and transmissibility of covid-19. Nat. Med..

[16] Johansson MA, Quandelacy TM, Kada S, Prasad PV, Steele M, Brooks JT, Slayton RB, Biggerstaff M, Butler JC (2021). SARS-CoV-2 transmission from people without covid-19 symptoms. JAMA network open.

[17] Althobaity, Y., Wu, J., Tildesley, M.J. A comparative analysis of epidemiological characteristics of mers-cov and SARS-CoV-2 in Saudi Arabia. *Infect. Dis. Model.* (2022). 10.1016/j.idm.2022.07.002 PMC9343745 35938094

[18] World Health Organization *et al.* Criteria for releasing covid-19 patients from isolation: scientific brief, 17 June 2020. Technical report, World Health Organization (2020).

[19] Prague, M., *et al.* Population modeling of early covid-19 epidemic dynamics in french regions and estimation of the lockdown impact on infection rate. medrxiv, 2020.04. 21.20073536. Google Scholar (2020).

[20] Riccio, A. Analysis of the SARS-CoV-2 epidemic in Lombardy (Italy) in its early phase. Are we going in the right direction? medRxiv (2020).

[21] Purkayastha S, Bhattacharyya R, Bhaduri R, Kundu R, Xuelin G, Salvatore M, Ray D, Mishra S, Mukherjee B (2021). A comparison of five epidemiological models for transmission of SARS-CoV-2 in India. BMC Infect. Dis..

[22] Plummer, M., Best, N., Cowles, K. & Vines, K. Package ‘coda’. http://cran.r-project.org/web/packages/coda/coda.pdf, accessed January 25:2015 (2015).

[23] Wang X, Pasco RF, Du Z, Petty M, Fox SJ, Galvani AP, Pignone M, Johnston SC, Meyers LA (2020). Impact of social distancing measures on coronavirus disease healthcare demand, Central Texas, USA. Emerg. Infect. Dis..

[24] Wells CR, Townsend JP, Pandey A, Moghadas SM, Krieger G, Singer B, McDonald RH, Fitzpatrick MC, Galvani AP (2021). Optimal covid-19 quarantine and testing strategies. Nat. Commun..

[25] Ding Z, Wang K, Shen M, Zhao S, Song W, Li R, Li Z, Wang L, Feng G, Zhiliang H (2021). Estimating the time interval between transmission generations and the presymptomatic period by contact tracing surveillance data from 31 provinces in the mainland of china. Fundam. Res..

[26] Li C, Zhu Y, Qi C, Liu L, Dandan Zhang X, Wang KS, Jia Y, Liu T, He D (2021). Estimating the prevalence of asymptomatic covid-19 cases and their contribution in transmission-using Henan province, China, as an example. Front. Med..

[27] Zeng Y, Guo X, Deng Q, Luo S, Zhang H (2020). Forecasting of covid-19: Spread with dynamic transmission rate. J. Saf. Sci. Resilience.

[28] Ghosal S, Bhattacharyya R, Majumder M (2020). Impact of complete lockdown on total infection and death rates: A hierarchical cluster analysis. Diabetes Metab. Syndrome Clin. Res. Rev..

[29] Sutton D, Fuchs K, D’alton M, Goffman D (2020). Universal screening for SARS-CoV-2 in women admitted for delivery. N. Engl. J. Med..

[30] Wang K, Lin Ding Yu, Yan CD, Minghan Q, Jiayi D, Hao X (2020). Modelling the initial epidemic trends of covid-19 in Italy, Spain, Germany, and France. PLoS ONE.

[31] Li R, Pei S, Chen B, Song Y, Zhang T, Yang W, Shaman J (2020). Substantial undocumented infection facilitates the rapid dissemination of novel coronavirus (SARS-CoV-2). Science.

[32] Grace, Y.Y., Hu, P. & He, W. Characterizing the dynamic of covid-19 with a new epidemic model: Susceptible-exposed-symptomatic-asymptomatic-active-removed. medRxiv (2020). 10.1002/cjs.11698 PMC9087003 35573897

[33] Khoshnaw SHA, Salih RH, Sulaimany S (2020). Mathematical modelling for coronavirus disease (covid-19) in predicting future behaviours and sensitivity analysis. Math. Model. Nat. Phenomena.

[34] Fernandez-Recio J (2020). Modelling the evolution of covid-19 in high-incidence European countries and regions: estimated number of infections and impact of past and future intervention measures. J. Clin. Med..

[35] Santamaría L, Hortal J (2020). Covid-19 effective reproduction number dropped during spain’s nationwide dropdown, then spiked at lower-incidence regions. Sci. Total Environ..

[36] Santamaría L, Hortal J (2020). Chasing the ghost of infection past: identifying thresholds of change during the covid-19 infection in Spain. Epidemiol. Infect..

[37] Baraniuk, C. Covid-19: How the UK vaccine rollout delivered success, so far. *BMJ* 372 (2021). 10.1136/bmj.n421 33602672

[38] Althobaity Y, Wu J, Tildesley MJ (2022). Non-pharmaceutical interventions and their relevance in the covid-19 vaccine rollout in Saudi Arabia and Arab Gulf countries. Infect. Dis. Model..

[39] Hatef E, Kitchen C, Chang H-Y, Kharrazi H, Tang W, Weiner JP (2021). Early relaxation of community mitigation policies and risk of covid-19 resurgence in the united states. Prevent. Med..

[40] Feldman AG, O’Leary ST, Danziger-Isakov L (2021). The risk of resurgence in vaccine-preventable infections due to coronavirus disease 2019-related gaps in immunization. Clin. Infect. Dis..

[41] Sungheetha A (2021). Covid-19 risk minimization decision making strategy using data-driven model. J. Inf. Technol..

