# Supplementary material for: Modelling the impact of non-pharmaceutical interventions on the spread of COVID-19 in Saudi Arabia
Source: Sci Rep. 2023 Jan 16;13:843. doi: 10.1038/s41598-022-26468-5 (PMC9842221; doi:10.1038/s41598-022-26468-5)
Supplement: Supplementary file 1 — Supplementary Information. [file 41598_2022_26468_MOESM1_ESM.pdf]

Modelling the impact of non-pharmaceutical interventions on the spread of COVID-19 in Saudi Arabia

Yehya Althobaity[1,2*], Michael J. Tildesley[1],

1 The Zeeman Institute for Systems Biology and Infectious Disease Epidemiology Research, School of Life Sciences and Mathematics Institute, University of Warwick, Coventry, United Kingdom

2 Department of Mathematics, Taif University, Taif, Kingdom of Saudi Arabia

* Corresponding Author: Yehya.Althobaity@warwick.ac.uk

Supplementary Materials

Parameter correlation and posterior sampling from model fitting

As seen in Figures S1, S2, S3, and S4, the only two parameters that remain substantially correlated are $\beta_{1,2}$ and $\gamma_{1,2}$. This is expected however, since if we sample a higher transmission rate $\beta_{1,2}$, the total number of infections would increase, necessitating a lower documented infection rate $\gamma_{1,2}$ to fit to the observed data. While $\beta_{1,2}$ and $\gamma_{1,2}$ are strongly correlated, it is critical to note that their posterior joint distributions are centralized in narrow parameter space, indicating that we have sufficient information to estimate these parameters. We demonstrated that our technique is capable of accurately estimating parameter values despite the high correlation between the MCMC samples of $\beta_{1,2}$, and $\gamma_{1,2}$.

In general, if the transition rate is high, the ascertainable infection rate will be high as well (i.e., if the transmission rate and the ascertainable infection rate are positively correlated), resulting in the same outputs. To maintain stable dynamics, a disease that spreads rapidly must be balanced with a disease that recovers rapidly. Increased system dynamics as a result of an infectious disease or a faster recovery implies that equivalent outputs will be observed sooner than with slower system dynamics since transition and recovery rates are negatively correlated to t [1].

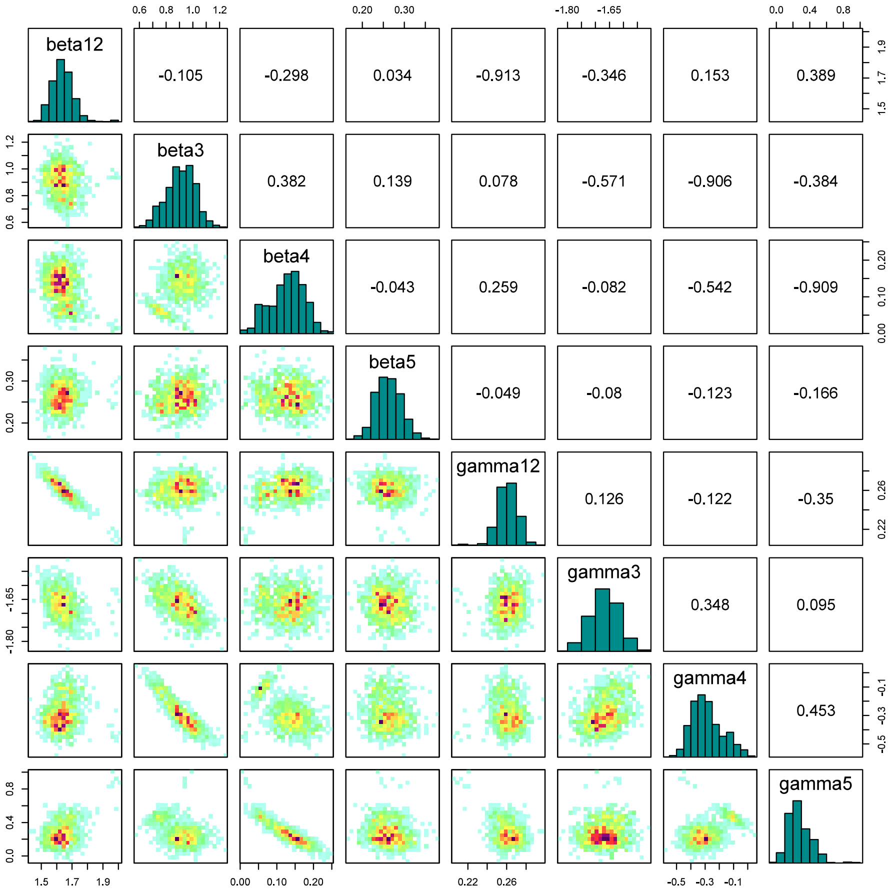

Figure S 1: Pairs plot of the Markov Chain Monte Carlo (MCMC) samples for Madinah region. The diagonal displays the marginal densities for the parameters. The upper triangle is Pearson correlations, meanwhile, the lower triangle shows scatter plots with different colours marking point density.

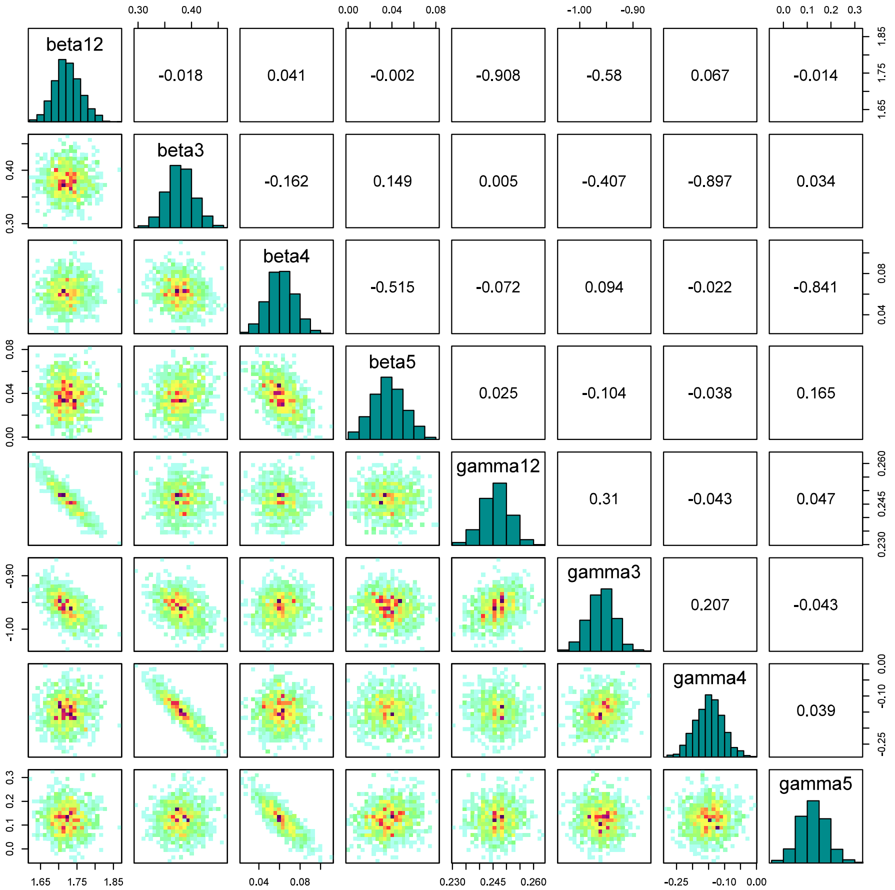

Figure S 2: Pairs plot of the Markov Chain Monte Carlo (MCMC) samples for Makkah region. The diagonal displays the marginal densities for the parameters. The upper triangle is Pearson correlations, meanwhile, the lower triangle shows scatter plots with different colours marking point density.

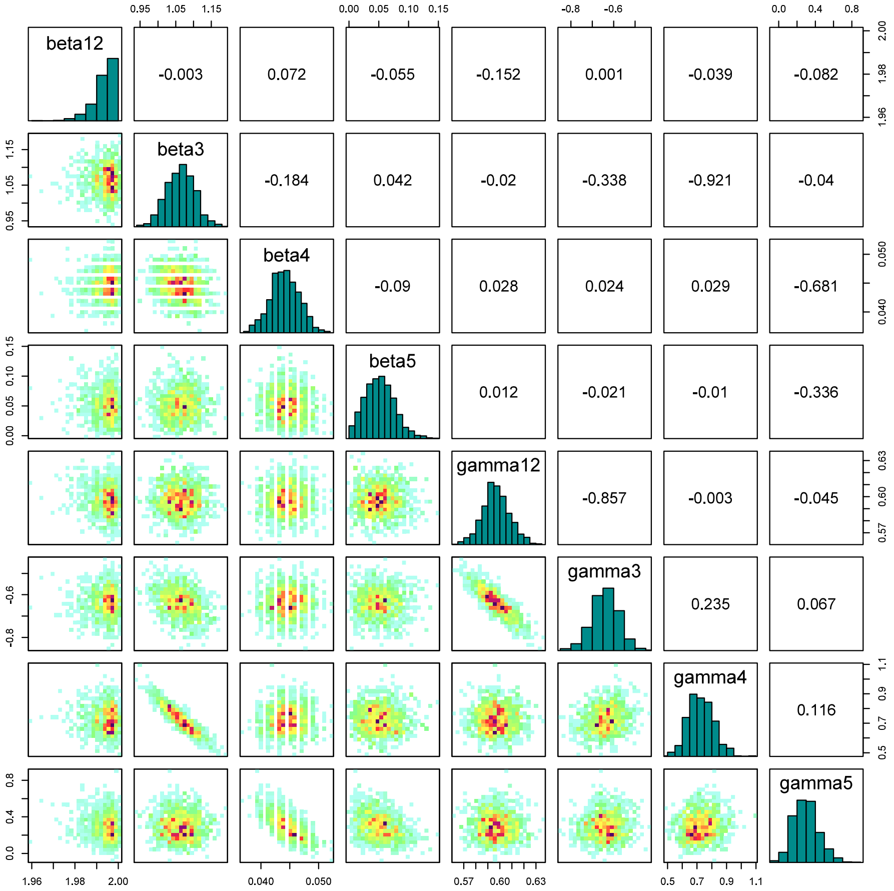

Figure S 3: Pairs plot of the Markov Chain Monte Carlo (MCMC) samples for the Eastern region. The diagonal displays the marginal densities for the parameters. The upper triangle is Pearson correlations, meanwhile, the lower triangle shows scatter plots with different colours marking point density.

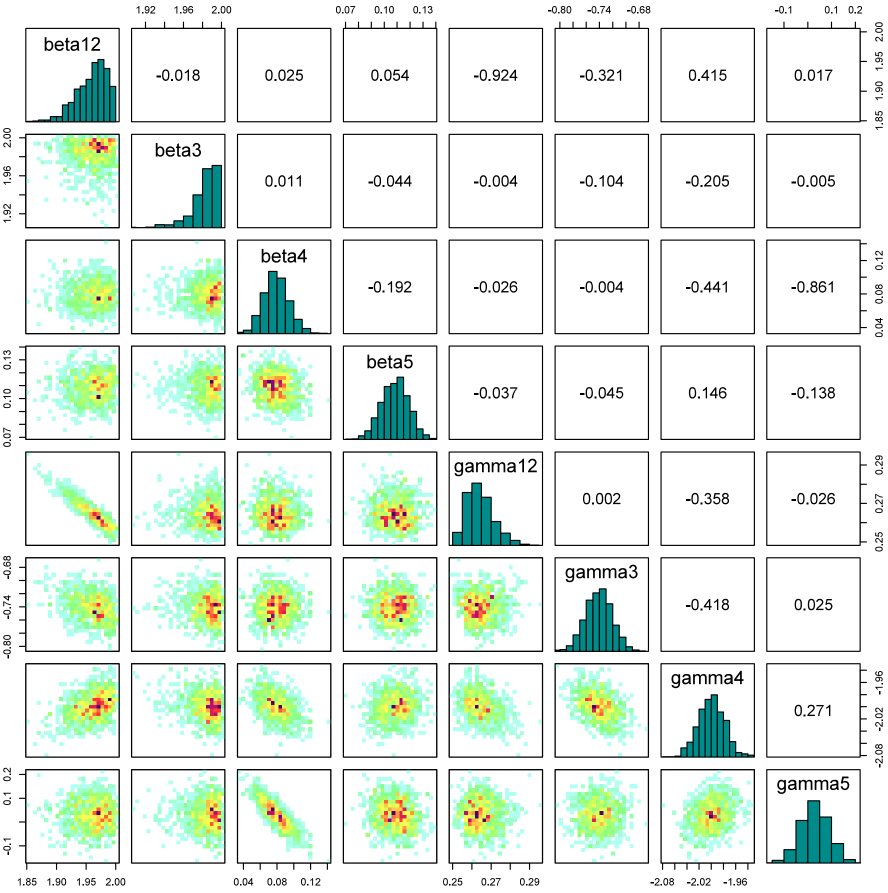

Figure S 4: Pairs plot of the Markov Chain Monte Carlo (MCMC) samples for Riyadh region. The diagonal displays the marginal densities for the parameters. The upper triangle is Pearson correlations, meanwhile, the lower triangle shows scatter plots with different colours marking point density.

Bibliography

[1] Karen Larson, Georgios Arampatzis, Clark Bowman, Zhizhong Chen, Panagiotis Hadjidoukas, Costas Papadimitriou, Petros Koumoutsakos, and Anastasios Matzavinos. Data-driven prediction and origin identification of epidemics in population networks. *Royal Society open science*, 8(1):200531, 2021.
